# Neurological Biomarker Profiles in Royal Canadian Air Force (RCAF) Pilots and Aircrew

**DOI:** 10.3390/brainsci14121296

**Published:** 2024-12-23

**Authors:** Shawn G. Rhind, Maria Y. Shiu, Oshin Vartanian, Shamus Allen, Miriam Palmer, Joel Ramirez, Fuqiang Gao, Christopher J. M. Scott, Meissa F. Homes, Gary Gray, Sandra E. Black, Joan Saary

**Affiliations:** 1 Defence Research and Development–Toronto Research Centre, Toronto, ON M3K 2C9, Canada; maria.shiu@ecn.forces.gc.ca (M.Y.S.); or oshinv1@mac.com (O.V.); 2 Faculty of Kinesiology and Physical Education, University of Toronto, Toronto, ON M5S 2W6, Canada; 3 Department of Psychology, University of Toronto, Toronto, ON M5S 3G3, Canada; 4 Canadian Forces Environmental Medicine Establishment, Toronto, ON M3K 2C9, Canada; shamus.allen@forces.gc.ca (S.A.); miriamspalmer@gmail.com (M.P.); gary.gray@forces.gc.ca (G.G.); or joan.saary@utoronto.ca (J.S.); 5 The Dr. Sandra Black Centre for Brain Resilience & Recovery, Sunnybrook Research Institute, Toronto, ON M4N 3M5, Canada or joelr@sri.utoronto.ca (J.R.); fgao@sri.utoronto.ca (F.G.); cscott@sri.utoronto.ca (C.J.M.S.); melissa.holmes@syneoshealth.com (M.F.H.); sandra.black@sunnybrook.ca (S.E.B.); 6 Graduate Department of Psychological Clinical Science, University of Toronto Scarborough, Toronto, ON M1C 1A4, Canada; 7 Department of Medicine, Division of Neurology, Sunnybrook Health Sciences Centre and University of Toronto, Toronto, ON M5S 3H2, Canada; 8 Department of Medicine, Division of Occupational Medicine, University of Toronto, Toronto, ON M5T 0A1, Canada

**Keywords:** aviation, high altitude, hypobaric decompression, blood biomarkers, neuron-specific enolase, glial fibrillary acidic protein, ubiquitin carboxyl-terminal hydrolase-L1, peroxiredoxin-6, neurofilament light chain, tau protein

## Abstract

Background/Objectives: Military aviators can be exposed to extreme physiological stressors, including decompression stress, G-forces, as well as intermittent hypoxia and/or hyperoxia, which may contribute to neurobiological dysfunction/damage. This study aimed to investigate the levels of neurological biomarkers in military aviators to assess the potential risk of long-term brain injury and neurodegeneration. Methods: This cross-sectional study involved 48 Canadian Armed Forces (CAF) aviators and 48 non-aviator CAF controls. Plasma samples were analyzed for biomarkers of glial activation (GFAP), axonal damage (NF-L, pNF-H), oxidative stress (PRDX-6), and neurodegeneration (T-tau), along with S100b, NSE, and UCHL-1. The biomarker concentrations were quantified using multiplexed immunoassays. Results: The aviators exhibited significantly elevated levels of GFAP, NF-L, PRDX-6, and T-tau compared to the CAF controls (*p* < 0.001), indicating increased glial activation, axonal injury, and oxidative stress. Trends toward higher levels of S100b, NSE, and UCHL-1 were observed but were not statistically significant. The elevated biomarker levels suggest cumulative brain damage, raising concerns about potential long-term neurological impairments. Conclusions: Military aviators are at increased risk for neurobiological injury, including glial and axonal damage, oxidative stress, and early neurodegeneration. These findings emphasize the importance of proactive monitoring and further research to understand the long-term impacts of high-altitude flight on brain health and to develop strategies for mitigating cognitive decline and neurodegenerative risks in this population.

## 1. Introduction

Military aviators, including pilots and aircrews, operate in high-stress environments that impose unique physiological challenges, such as hypobaric hypoxia, decompression stress, and exposure to high gravito-inertial (G) forces [1,2,3]. These stressors, stemming from reduced atmospheric pressure and fluctuating oxygen levels, can disrupt cerebral blood flow, resulting in brain hypoxia and hypoperfusion [4,5,6]. Repeated exposure to these conditions has been associated with cognitive deficits, including impairments in processing speed, memory, and attention, raising concerns about their long-term impact on neurological health [7,8,9,10]. Despite advancements in aircraft pressurization systems, nonhypoxic hypobaria and decompression stress remain significant concerns, particularly for military aviators operating in extreme scenarios such as U-2 reconnaissance missions and high-speed fighter jet flights [11,12,13,14]. These cumulative stressors strain the brain and central nervous system (CNS), heightening the risk of both acute and chronic neurological injury [15,16].

Rapid barometric pressure changes inherent in high-altitude aviation can trigger decompression sickness (DCS) and microbubble formation, leading to microvascular damage and disruption of the blood–brain barrier (BBB) [17,18,19]. Circulating microparticles, released by activated or injured cells, are thought to contribute to BBB permeability, mediate inflammatory responses, and promote neuroinflammation [20,21,22]. Compromised BBB integrity permits the influx of inflammatory mediators and immune cells into the brain, fueling chronic neuroinflammation, neuronal damage, and cognitive decline [23,24,25]. Hypobaric hypoxia and oxidative stress further exacerbate microstructural brain damage, often presenting as white matter hyperintensities (WMHs) on MRI scans. WMHs, associated with deficits in memory, executive function, and attention, have been observed even in asymptomatic individuals, underscoring concerns about the long-term neurological health of aviators [21,26,27,28,29,30,31,32,33,34].

In addition to hypoxia, high G-forces during flight amplify brain hypoperfusion and oxidative stress, disrupting cellular metabolism and promoting the production of pathological proteins such as phosphorylated tau, a marker implicated in neurodegenerative processes [35,36,37,38]. These combined stressors create a high-risk environment for progressive neurological injury, necessitating a deeper understanding of the underlying mechanisms of brain damage in this population [39,40,41].

Blood-based neurological biomarkers, including S100b, neuron-specific enolase (NSE), glial fibrillary acidic protein (GFAP), neurofilament light chain (NF-L), phosphorylated neurofilament heavy (pNF-H), ubiquitin carboxyl-terminal hydrolase (UCH-L1), peroxiredoxin-6 (PRDX-6), and microtubule-associated total protein tau (T-tau), offer critical insights into the biological processes underpinning brain injury [42,43,44]. Elevated levels of these biomarkers reflect glial activation, neuronal and axonal damage, BBB disruption, and neuroinflammation [23,45]. Specifically, S100b and GFAP signal glial and astrocytic injury; NSE, UCH-L1, NF-L, and pNF-H indicate neuronal and axonal degeneration; PRDX-6 reflects oxidative stress; and T-tau serves as a marker of ongoing neurodegeneration [46,47,48,49]. These biomarkers provide a valuable tool for early detection of brain injury and the development of targeted strategies to mitigate the neurological effects in populations exposed to high-altitude aerospace environments [44].

Despite advancements in aviation technology, the persistence of occupational hazards such as nonhypoxic hypobaria and decompression stress continues to pose significant neurological risks to military aviators [50]. The association between WMHs and elevated biomarker levels suggests potential long-term cognitive and neurological impairments [51]. Addressing these risks requires ongoing research to elucidate the injury mechanisms and develop effective prevention and intervention strategies to safeguard the brain health and operational readiness of military personnel [21,52].

This study evaluated the impact of high-performance flight environments—characterized by decompression stress, G-forces, and intermittent hypoxia—on blood-based biomarkers in Royal Canadian Armed Forces (RCAF) pilots and aircrew members compared to unexposed controls. The findings revealed significant elevations in key biomarkers, including GFAP, NF-L, PRDX-6, and T-tau, indicative of increased risks of glial activation, axonal damage, oxidative stress, and early neurodegeneration. This work represents the first investigation of these biomarkers in Canadian military aviators, underscoring the importance of ongoing monitoring and the development of targeted protective strategies to preserve the brain health of this at-risk population.

## 2. Materials and Methods

### 2.1. Study Participants

The study included 48 RCAF aviators with extensive high-performance flight experience and 48 age- and sex-matched controls without similar occupational exposure (Table 1). Participants were all non-smoking males, with a mean age of 39 years. Controls were selected to match general health and lifestyle variables, without significant high-performance flight exposure.

### 2.2. Experimental Design and Procedures

This cross-sectional study of blood-based neurological injury biomarker profiles in RCAF aviators is part of the larger “Canadian White Matter Hyperintensity Study in Canadian Armed Forces (CAF) Aviators”, which investigates the effects of occupational aviation stressors on neurological health [53,54]. Ethical approval was obtained from the Defence Research and Development Canada (DRDC) Human Research Ethics Committee (HREC No. 2018-051).

Data collection occurred over two days at the Canadian Forces Environmental Medicine Establishment (CFEME) and DRDC Toronto Research Centre (TRC). Participants provided demographic information and completed detailed health histories, including flight and exposure data, prior concussions or brain injuries, and a comprehensive battery of neuropsychological and physiological performance tests. All participants refrained from decompression exposure for at least 48 h and from strenuous activity for 24 h before testing. To ensure consistency, all testing occurred at the same time of day. The multidimensional dataset was de-identified and entered into a central database for analysis, enabling a robust examination of the neurological effects of high-altitude occupational exposures in military aviators.

### 2.3. Blood Collection, Processing, and Storage

Peripheral blood was collected from consenting volunteers in a fasting state by a trained technologist using standard phlebotomy techniques. Venous blood samples were drawn into 10 mL K_2_EDTA tubes (BD Vacutainer^®^, Franklin Lakes, NJ, USA), immediately centrifuged at 1600× *g* for 15 min at 4 °C, separated into plasma aliquots, and stored at −80 °C until analysis. All samples were processed in the same manner at the same time of day.

### 2.4. Plasma Neurological Biomarker Analyses

Plasma samples were analyzed for a panel of eight neuroproteomic biomarkers, selected on the basis of their relative brain specificities and potentials to reflect distinct pathoanatomical injury mechanisms, including (1) astroglial damage/gliosis (S100b, GFAP); (2) neuronal cell body damage (NSE, UCH-L1); (3) diffuse axonal injury (NF-L, pNF-H); (4) cerebral oxidative stress PRDX-6; and (5) neurodegeneration (T-tau). Total plasma concentrations of each molecule were quantified using a combination of ultra-sensitive multiplexed neurological immunoassay panels from MesoScale Diagnostics, LLC (MSD^®^, Gaithersburg, MD, USA), Multi-Array^TM^ Human Electrochemiluminescence assays, and Single Molecule Array (SiMoA^TM^) Human Neurology 4-Plex assays (Quanterix^®^, Lexington, MA, USA), as previously reported [55,56,57,58]. Duplicates were used for intra-assay and inter-assay precision testing. To minimize assay variations, all specimens were analyzed on the same day, in duplicate, in random order, by a technician who was blind to the participants’ status.

### 2.5. Statistical Analysis

Biomarker data were considered usable if they fell within the detection limits specified by the assay manufacturer and exhibited a coefficient of variation (CV) of less than 15% between duplicate measurements. Plasma biomarker concentrations are reported as medians with interquartile ranges (IQRs) to account for potential non-normal distributions. Group differences in biomarker levels between aviators and controls were assessed using the Mann–Whitney *U*-test, with false discovery rate (FDR) correction applied to control for multiple comparisons. Participant demographic and occupational characteristics were evaluated using independent two-sample *t*-tests for continuous variables and chi-squared (Χ^2^) tests for categorical variables. All statistical analyses were conducted using GraphPad Prism (v10.2.3), with significance set at *p* < 0.05 (FDR-adjusted where applicable).

## 3. Results

### 3.1. Participant Characteristics

All the participants were non-smokers, and the aviators and controls were closely matched with respect to age, sex, BMI, and general health (Table 1). The aviators, however, reported significantly greater cumulative flight hours and more frequent exposure to high-performance flight conditions than the controls.

### 3.2. Blood Neurological Biomarker Profiles

**Figure 1:**
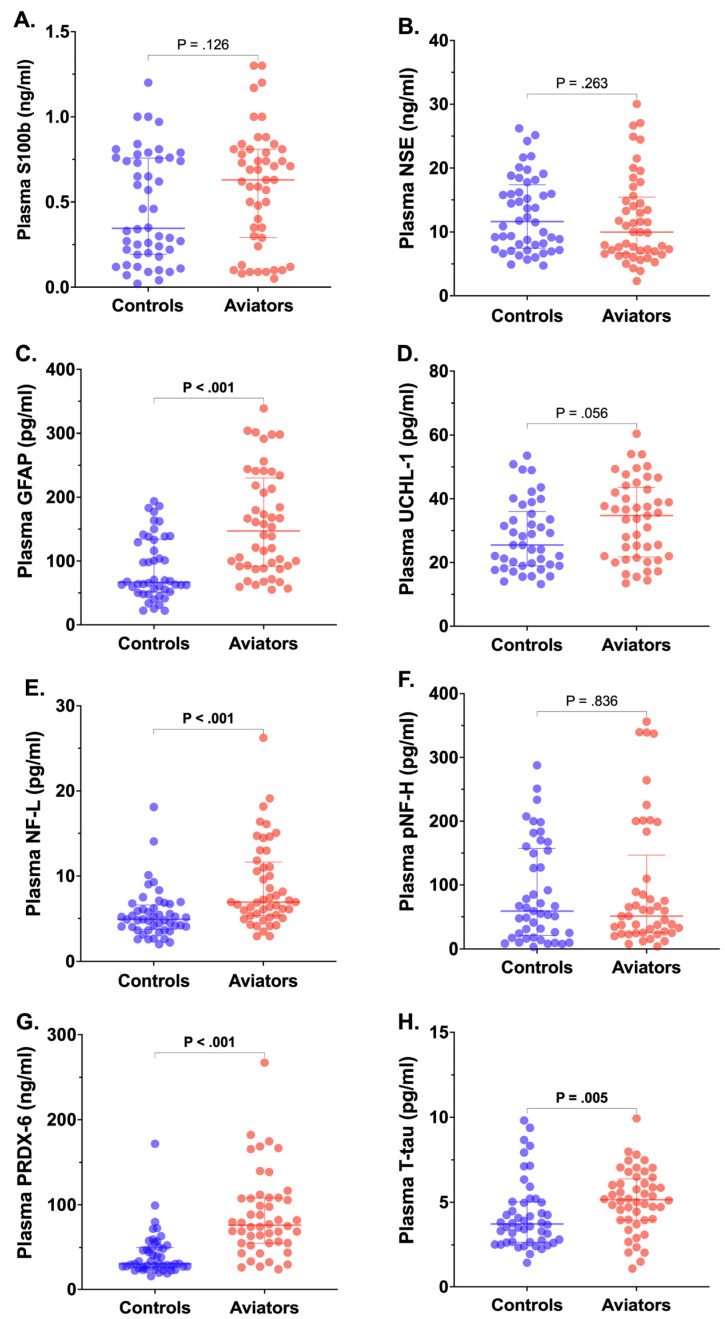
Neurological biomarker profiles in plasma from CAF *aviators* (pilots and flight crew; red dots, *n* = 48) versus healthy *controls* (blue dots, *n* = 48), plotted for S100b (**A**), neuron-specific enolase (NSE; (**B**)), glial fibrillary acidic protein (GFAP; (**C**)), ubiquitin carboxy-terminal hydrolase L1 (UCH-L1; (**D**)), neurofilament light (NF-L; (**E**)), phosphorylated neurofilament heavy (pNF-H; (**F**)), peroxiredoxin 6 (PRDX-6; (**G**)), and total tau (T-tau; (**H**)). Each dot represents the biomarker concentration (as indicated) for an individual subject; solid lines show medians with interquartile ranges. Significant group differences (*p* < 0.05) in biomarker values by *Mann–Whitney U-test* are displayed for each marker, corrected for multiple comparisons at FDR = 0.05.

The aviators exhibited significant elevations in the plasma levels of GFAP, NF-L, PRDX-6, and T-tau compared to the controls (Figure 1), indicating glial activation, axonal damage, oxidative stress, and potential neurodegeneration. While S100b, a marker of brain injury and BBB disruption, did not show a statistically significant difference between the groups (*p* = 0.126), there was a trend toward higher levels in the aviators. Similarly, NSE, a marker of neuronal injury, was not significantly different between the groups (*p* = 0.263), reflecting comparable levels of overt neuronal damage.

In contrast, GFAP, a marker of astrocytic injury and reactivity, was significantly elevated in the aviators (*p* < 0.001), suggesting that these individuals may be experiencing heightened astrocyte activation, potentially due to repeated exposure to hypobaric conditions or mild hypoxia during high-altitude flights. UCHL-1, another neuronal injury marker, showed a trend toward higher levels in the aviators, although it did not reach statistical significance (*p* = 0.056), potentially reflecting early-stage or subclinical neuronal stress.

Notably, NF-L, a well-established biomarker of axonal damage, was significantly higher in the aviators compared to the controls (*p* < 0.001), providing strong evidence of increased axonal injury in this group. However, the levels of pNF-H, another axonal damage marker, did not differ significantly between the groups (*p* = 0.836), suggesting that specific types of axonal injury may be more pronounced than others in the aviators. Additionally, PRDX-6, a key antioxidant enzyme involved in cellular protection from oxidative stress, was significantly elevated in the aviators (*p* < 0.001), indicating a greater oxidative stress burden, likely due to the environmental challenges and oxidative damage associated with high-altitude flight.

Lastly, plasma T-tau, a marker of neurodegeneration, was modestly but significantly elevated in the aviators compared to the controls (*p* = 0.005). Elevated tau levels are associated with neurodegenerative diseases, and this finding may reflect chronic low-level neuronal injury linked to the occupational exposures experienced by military aviators, including decompression stress and intermittent hypoxia.

## 4. Discussion

This observational cohort study assessed the blood-based neurological biomarkers (S100b, NSE, GFAP, NF-L, pNF-H, PRDX-6, and T-tau) in military aviators exposed to hypobaria, hypoxia, and G-forces. The results reinforce prior evidence that pilots and flight crews face elevated risks of neurological injury from repeated aviation-specific stressors, including decompression and intermittent low-level hypoxia. Significant elevations in those biomarkers linked to glial activation, axonal damage, and oxidative stress are consistent with previous studies demonstrating that aerospace environments can induce chronic neuroinflammation, brain structural changes, and cognitive deficits.

### 4.1. Astrocyte Activation and Neuroinflammation

The elevated GFAP in aviators suggests astrocyte activation, a hallmark of neuroinflammatory conditions and brain injury [59,60]. Growing evidence supports the clinical utility of blood GFAP levels as a biomarker for neuroinflammatory and neurodegenerative diseases, as well as CNS involvement in systemic conditions [61,62]. Our findings align with those of Abou-Donia et al., who found that a flight crew demonstrated increased CNS-specific serum autoantibodies indicative of neuronal injury and gliosis linked to environmental exposures [63]. While astrocytes are essential for maintaining brain homeostasis, chronic activation due to injury or stress is a known contributor to neurodegenerative diseases like Alzheimer’s and other tauopathies [60,64,65]. These results indicate that repeated exposure to decompression stress and changes in gravitational force may lead to subtle BBB disruption, triggering sustained neuroinflammation and increasing the risk of long-term neurological complications [2,66]. Similar GFAP elevations have been observed in individuals exposed to high-altitude hypoxia and mild brain injuries, further supporting the link between flight stressors and neuroinflammation [67,68,69].

### 4.2. Axonal Damage and White Matter Changes

NF-L is a key cytoplasmic structural protein that forms complexes with other neurofilament proteins, such as NF-H, and plays a crucial role in maintaining the integrity and function of the neuronal cytoskeleton, particularly in large myelinated axons abundant in subcortical white matter tracts [70,71]. These axons facilitate rapid communication between brain regions [72,73]. Elevated NF-L levels, as observed in our study, are consistent with the findings in other at-risk populations, such as high-impact athletes and military personnel exposed to blast overpressure, where axonal damage correlates with cognitive dysfunction [58,74,75]. When axons are damaged due to trauma, neurodegeneration, or mechanical stress, NF-L is released into the bloodstream, serving as a sensitive biomarker of neuronal injury [49,76,77]. Increased NF-L levels in biofluids are strongly indicative of axonal damage, reflecting the extent of injury and signaling ongoing neurodegenerative or traumatic processes [73,78,79]. These elevations are often linked to white matter pathway damage, as reflected in MRI findings as WMHs [80,81].

Structural neuroimaging studies consistently reveal increased prevalence of WMHs and microstructural brain changes in military pilots and flight crews, suggesting that axonal injury is a frequent consequence of high-altitude flight [9,32,33,39,82,83,84]. Although WMHs are typically associated with aging and small-vessel cerebrovascular disease, they occur more frequently in military aviators, even in the absence of DCS or overt brain injury symptoms [9,50]. The elevated levels of NF-L detected in these aviators likely stem from a combination of rapid barometric pressure changes, microbubble formation during decompression, and the high G-forces experienced during flight [19,21,50].

Studies of U-2 pilots exposed to extreme nonhypoxic hypobaric and decompression stress exhibited significantly higher volumes and numbers of WMHs compared to non-pilot controls [26,28,29,30,31]. These microstructural brain changes were linked to cognitive performance deficits, particularly in attention, processing speed, working memory, and executive function [30,32]. The increased WMH burden mirrors the symptoms observed in brain injury and other neurological disorders, where reduced axonal integrity is closely associated with a heightened risk of developing mild cognitive impairment and dementia [85,86,87]. These results, along with similar findings in mountaineers, astronauts, and divers, underscore the link between high-altitude exposures and axonal integrity [88,89,90,91].

Elevated circulating NF-L levels are similarly correlated with cognitive decline and the presence of WMHs in conditions such as aging, cerebral small vessel disease, and neurodegeneration [85,92]. Research shows that NF-L not only reflects an existing brain pathology but also serves as a predictor of future cognitive decline and neurodegenerative disorders [71,93,94]. The elevated NF-L levels observed in our study provide critical biomolecular evidence supporting prior imaging findings, indicating ongoing axonal damage that may predispose military aviators to long-term cognitive decline and neurological disorders due to repeated high-altitude exposure [21,81,95].

### 4.3. Upregulation of PRDX-6 with Oxidative Stress and Brain Injury

PRDX-6 has emerged as a key biomarker for neurological injury due to its integral role in mitigating oxidative stress, a major driver of neuronal and white matter damage [55,96]. Primarily expressed by astrocytes in the CNS, PRDX-6 functions as both a glutathione peroxidase and a phospholipase A2, neutralizing harmful reactive oxygen species (ROS) and repairing oxidatively damaged lipid membranes [97,98]. These functions are critical for maintaining neuronal integrity as neurons are particularly vulnerable to ROS-driven oxidative stress, especially under hypoxic conditions, due to their high metabolic demands [99,100].

Elevated PRDX-6 levels in military aviators likely reflect this increased oxidative burden, driven by intermittent hypoxia, decompression stress, and high G-forces during flight [9]. Hypoxia-induced reductions in oxygen levels trigger an overproduction of ROS, leading to damage to DNA, proteins, and lipids [101], while subsequent reoxygenation further exacerbates oxidative stress [102]. This sustained oxidative environment can overwhelm the brain’s natural antioxidant defenses, increasing the risk of cognitive decline, neuroinflammation, and neurodegenerative diseases [103,104,105]. PRDX-6 plays a critical role in these high-risk conditions by neutralizing peroxides and repairing oxidatively damaged cell membranes, offering a protective mechanism against the chronic oxidative stress associated with repeated high-altitude exposure [37,106].

PRDX-6 serves as a critical biomarker for oxidative stress and neurological injury, offering valuable insights into the physiological challenges faced by military aviators [56,58]. Its upregulation aligns with findings in other hypoxic populations, such as mountaineers and astronauts, where increased antioxidant activity acts as a protective mechanism against oxidative damage [99,106,107,108,109]. Specifically, PRDX-6 mitigates oxidative stress by neutralizing ROS and repairing oxidatively damaged cell membranes [99,106,107,108,109]. This dual functionality positions PRDX-6 as a vital adaptive response to both hypoxia, experienced during high-altitude flights, and hyperoxia, encountered in military operations involving supplemental oxygen or hyperbaric oxygen therapy [22,36,110,111,112]. The elevated PRDX-6 levels in aviators highlight the oxidative stress burden associated with repeated exposure to hypoxia, decompression stress, and high G-forces, underscoring its potential for monitoring brain health [22]. Future studies should further explore PRDX-6’s role in optimizing oxygen exposure protocols and developing protective strategies for high-risk aviation environments [3,44].

### 4.4. Elevated Tau and Risk of Neurodegeneration in Aviators

Tau is a microtubule-associated protein primarily found in unmyelinated cortical axons, where it stabilizes the cytoskeleton and maintains the neuronal structure [113]. Under pathological conditions, tau can become hyperphosphorylated, detach from microtubules, and form neurofibrillary tangles—a hallmark of tauopathies such as Alzheimer’s disease and chronic traumatic encephalopathy (CTE) [114,115]. Alongside tau pathology, activated microglia, astrocytes, and elevated proinflammatory mediators are found in those brain regions affected by neurodegeneration [64,116]. While tauopathies are commonly associated with repeated head impacts [117], military aviators are exposed to environmental stressors that may similarly promote tau accumulation [50,118,119].

In our study, the military aviators exhibited modestly elevated T-tau levels compared to the non-aviators, suggesting that occupational stressors such as decompression, intermittent hypoxia, and/or high G-forces may contribute to neuronal damage [9,95]. Even small increases in peripheral tau are associated with tau aggregation in the brain, cognitive decline, and neurodegenerative dementias [49,64]. Elevated tau in aviators may indicate early neurodegeneration or chronic neuroinflammation [116,120]. Furthermore, chronic intermittent hypobaric hypoxia, possibly experienced during some high-altitude flights, disrupts brain metabolism, induces oxidative stress, and promotes tau hyperphosphorylation, potentially leading to synaptic dysfunction, cognitive impairment, and increased risk of tauopathies like CTE [121,122,123].

Our findings align with those of Iacona et al., who demonstrated in swine models that high-altitude hypobaric conditions lead to increased phosphorylated tau in the hippocampus, along with astroglial and microglial activation, indicating neuroinflammation [38]. They also observed myelin loss in the hippocampus and cerebellum, suggesting cognitive and motor impairments. Similarly, Abou-Donia et al. reported elevated serum autoantibodies to tau in aircrew members with neurological symptoms, and Rosén et al. found significant increases in blood tau levels after diving, indicating neuronal damage due to decompression stress [124]. These studies mirror our findings in aviators, where modestly elevated tau levels suggest that repeated aviation-related stressors may contribute to early neuronal injury and tau accumulation. Together, these results underscore the shared risks of tauopathies in military aviators and high-altitude workers [41,124,125].

Aviators with a history of brain injury, concussion, or trauma face even greater risks [126]. Brain injuries disrupt the BBB, facilitating abnormal tau metabolism, while flight conditions exacerbate pre-existing damage; hypoxia, G-forces, and decompression increase oxidative stress, intracranial pressure, and impair blood flow, contributing to tau aggregation [121,127,128]. This “double-hit” scenario may accelerate tau pathology, particularly in individuals with repeated head trauma [129,130]. Research shows that those with prior brain injuries are more vulnerable to neurodegenerative diseases like CTE when exposed to repetitive stressors, reinforcing the connection between elevated tau and long-term neurodegeneration risks in aviators [50,115].

These findings raise concerns regarding the long-term brain health of military aviators and emphasize the need for further research on the impact of high-altitude flight in terms of tau-related neurodegeneration. Proactive monitoring of tau levels could provide early indicators of neurodegenerative conditions, enabling timely intervention to mitigate the neurological risks faced by military aviators [3,21,44].

### 4.5. S100b, NSE, and UCHL-1 Trends

Although the S100b and NSE levels were not significantly elevated in this study, the observed trends toward higher levels in the aviators remain noteworthy. S100b, a well-established marker of BBB dysfunction, has previously been linked to hypoxic exposure in aviators. While no statistically significant increases were detected, the upward trend may indicate subclinical BBB disruption, which could become more pronounced with prolonged exposure or among individuals with extensive flight histories. Similarly, NSE and UCH-L1, markers of neuronal injury, are known to increase in response to mechanical stress and potential microemboli from gas bubbles. The subtle elevation in UCH-L1 levels observed in this cohort may reflect ongoing low-grade neuronal damage that, although not acute, could contribute to cumulative brain injury over time. These findings emphasize the need for further longitudinal research to assess the implications of these subtle yet potentially impactful biomarker changes.

### 4.6. Implications for Cognitive Function and Long-Term Risks

Elevations in GFAP, NF-L, and PRDX-6 point to a cycle of BBB disruption, axonal injury, oxidative stress, and neuroinflammation—factors that are closely linked to cognitive decline and neurodegenerative diseases [131,132]. Previous studies have shown that individuals exposed to both hypobaric decompression stress during flight and chronic high-altitude conditions may experience subtle decrements in cognitive performance, particularly in attention, memory, and executive function in association with WMHs [30,133,134]. These biomarker changes raise concerns regarding long-term neurological conditions, including CTE and early dementia [119,135], as documented in other populations exposed to repetitive neurotrauma [115,123].

### 4.7. Study Limitations

Several limitations should be noted when interpreting these findings. First, the cross-sectional design provides a snapshot of the biomarker levels at a single time point, limiting the ability to establish causal relationships or assess the progression of long-term neurological changes in aviators. Longitudinal studies are needed to determine whether biomarker elevations, such as those observed here, predict future neurodegenerative conditions. Second, while biomarkers like GFAP and NF-L are indicative of glial and axonal injury, they lack specificity for aviation-related stressors and may be influenced by other factors, including prior concussions or physical stressors. Third, the lack of direct correlations between the biomarker levels and cognitive performance data limits the ability to link any biological changes to functional impairments. Future studies incorporating cognitive assessments will provide a more comprehensive understanding of these associations. Fourth, blood-based biomarkers primarily reflect peripheral levels and may not fully capture a brain-specific injury due to limited blood–brain barrier permeability and the potential impact of glymphatic clearance. Moreover, markers such as NSE, GFAP, and S100b are expressed in peripheral tissues, complicating the interpretation of their elevations. Fifth, the lack of well-established clinical reference ranges for mild or subclinical injuries creates challenges in determining whether modest biomarker increases signify pathological changes. Lastly, this study did not account for variability in flight hours, altitude exposure, or oxygen use, all of which could significantly influence decompression and hypoxic stress. Future research should stratify participants based on these variables to better understand their effects on brain injury biomarkers and the associated outcomes.

## 5. Conclusions

This study provides evidence that military personnel are at heightened risk of neurobiological dysfunction and damage due to their occupational exposures. The observed elevations in key brain biomarkers—GFAP, NF-L, tau, and PRDX-6—suggest significant glial activation, axonal injury, and oxidative stress, all of which pose potential long-term threats to brain health. While markers such as S100b, NSE, and UCHL-1 exhibited non-significant trends, their association with white matter hyperintensities and increased neuroinflammation and neurodegeneration markers underscores the concerns regarding cumulative brain injury in this population. These findings raise critical questions regarding the potential for long-term neurological impairments, cognitive decline, and the development of neurodegenerative diseases, including tauopathies, in military pilots and flight crews. Addressing these risks will require further research with larger cohorts, longitudinal designs, and precise exposure assessments. Proactive strategies for monitoring structural and molecular brain injury markers are essential to safeguard the cognitive health, operational readiness, and long-term well-being of military aviators.

## Figures and Tables

**Table 1:** Demographic, physical, and military occupational characteristics of participants.

Variable ^1^	Aviators (*n* = 48)	Controls (*n* = 48)	*p*-Value *
Age, years	39.0 ± 11.9	35.7 ± 6.3	0.074
Sex, *n* (%) male	48 (100%)	48 (100%)	–
Mass, kg	85.1 ± 9.1	83.8. ± 7.4	0.444
BMI, kg/m^2^	26.8 ± 2.9	25.4 ± 3.1	0.515
Total Flight Time, hours	2638.6 ± 376.0	–	–
Annual Flight Time, hours	106.7 ± 83.1	–	–
High Performance Flight, hours	2088.2 ± 282.7	–	–
Concussion, *n* (%)	3 (6.2)	2 (4.2)	0.416

^1^ Values are mean (±SD) or *n* (%) unless otherwise indicated; * independent two-sample *t*-test or chi-squared (Χ^2^) test for aviators vs. controls for continuous or categorical data, respectively. BMI = body mass index.

## Data Availability

The datasets presented in this article are not publicly available because they contain information that could compromise the privacy of military research participants. Requests to access the datasets should be directed to corresponding author.
